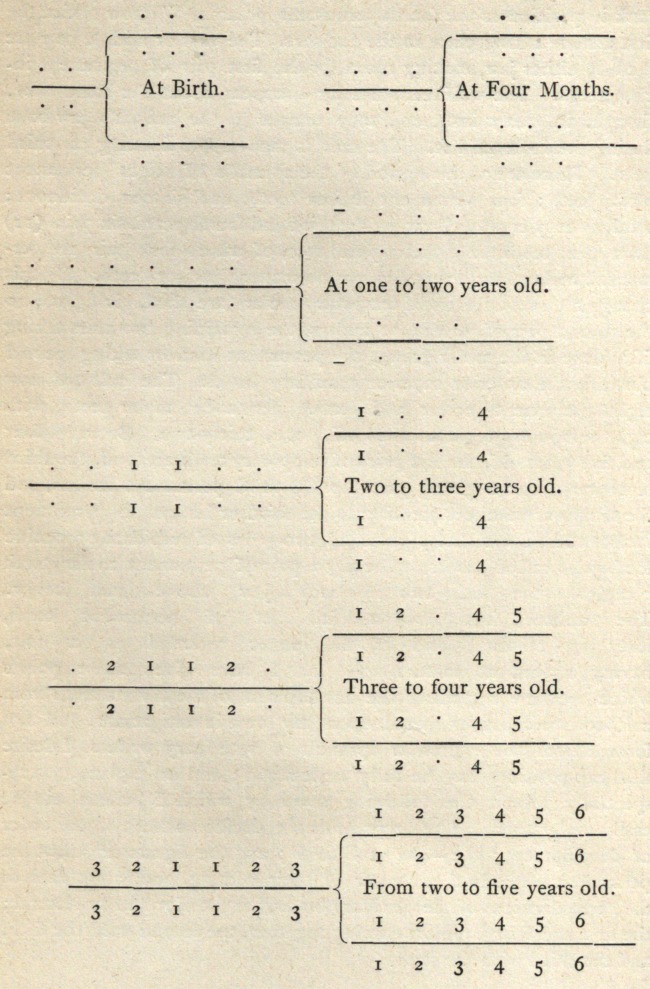# The Teeth of the Horse and Their Diseases

**Published:** 1882-10

**Authors:** R. Jennings


### ART. II.—THE TEETH OF THE HORSE AND THEIR DISEASES.

(*Continued.*)

BY R. JENNINGS, V.S.

The wolf teeth are last of the deciduous group to make their appearance in the mouth, usually between the first and second year. At this period the colt usually has a full mouth, or twenty-six deciduous or temporary teeth. A very important change is now going on in the jaws of the animal, which for simplicity of description the anatomical rule of writers on human anatomy will be observed. The reader will understand the term superior, ("upper, higher, denoting the relative position,") as relating to the upper jaw or superior maxillary. Inferior has the same reference to the lower jaw or inferior maxillary bone. The teeth of most animals offer some criterion by which to estimate with some degree of accuracy their age. This is chiefly accomplished by observing the natural changes in nature's onward course, which take place in the masticators; the periods at which they appear, are shed and replaced, and the alterations in their form and markings. In the ox the horns present legible indications of the advancing years. Reference to the teeth as indicating the age of the horse, is of ancient origin, as the writings of Xenophon, Hippocrates, and others from among the Greeks, and by several authors among the Romans, fully testify. In the selection of cavalry horses for the Grecian armies, all those having lost their dental marks were rejected. These facts were noticed by Varro, Columella, Vegetius, and other authors of more recent date, whose names live in history. We have now arrived at that period in the animal's life where the changes in the teeth mark with much certainty the age of the animal. While these positive indications of age are steadily going on, nature's attentive student will experience little difficulty in deciding the approximate age of the animal examined. The first notable change taking place in the development of the permanent teeth is the shedding or throwing off the first two pairs of the temporary molar teeth, one pair in the superior, and one pair in the inferior maxillary bones, which are replaced by the first pair of permanent molar teeth in either jaw, filling up the alveolar or tooth cavity so recently occupied by these transitory visitors; with these come a second pair of permanent molar teeth taking their place, posteriorily to the third temporary molar teeth in either jaw. These rank in position as the fourth permanent molars. We now notice the first change taking place in the incisors. The first or middle pair are shed in either jaw, making room for the first pair of permanent incisor teeth. The wolf teeth usually disappear about the same time, though, in many instances, they remain in the animal's jaw-bone during their natural life. The colt is now approximating its third year. There are at this period of the animal's life eight permanent molar teeth; four permanent incisor teeth, and sixteen deciduous; total, twenty-eight. Between the third and fourth years, six pairs (12) permanent teeth make their appearance, of which four pairs are permanent molars, and two pairs are permanent incisor teeth. By this change the second pairs of temporary molars are shed, giving way to the second pair of permanent molars, the remaining two pairs taking fifth place in the molar group, the permanent incisors taking second place in the anterior superior maxillary bones. The colt has now eight permanent incisor and sixteen permanent molar teeth, with eight temporary teeth to shed off; total, thirty-two. Between four and five years old, the last pairs of temporary teeth are shed, the third temporary molars giving place to the third permanent molars, and the temporary incisors yielding to the third or last pair of permanent incisor teeth; with these come up two pairs of cuspidata or canine teeth, also called tushes. The sixth pair of permanent molar teeth complete the set, two in each jaw above and below. The animal has now a full set of permanent teeth, forty in number, and is no longer regarded as a colt, having arrived at mature age. As I have given some points which are new regarding the development of the horse's teeth from colthood to maturity, based upon my own investigations, and not formed from the expressed opinions of veterinary writers, I desire that my position may be fully understood; and in explanation, respectfully submit the following summary, which I believe will be easily understood. The . represents the decidious teeth in the order of development; the – the wolf teeth; and the figures represent the permanent teeth, in the manner of their coming, one pair in each of the three divisions of the maxillary bones, the anterior and two lateral; the first and fourth molars coming together, the second with the fifth, and the third with the sixth.

Deciduous teeth at birth or within a few days:

. . .
———
. . .
. . ——— At Birth.
. .
. . .
———
. . .

. . .
———
. . .
. . . . ——— At Four Months.
. . . .
. . .
———
. . .

– . . .
———
. . .
. . . . . . ——— At one to two years old.
. . . . . .
. . .
———
– . . .

I . . 4

———

I . . 4

. . I I . . ——— Two to three years old.

. . I I . .

I . . 4

———

I . . 4

I 2 . 4 5
———
I 2 . 4 5
. 2 I I 2 . ——— Three to four years old.
. 2 I I 2 .
I 2 . 4 5
———
I 2 . 4 5

I 2 3 4 5 6

———

I 2 3 4 5 6

3 2 I I 2 3 ——— From two to five years old.

3 2 I I 2 3

I 2 3 4 5 6

———

I 2 3 4 5 6

The permanent incisor teeth are narrow from front to back, and broad from side to side upon their upper surface, with an oblong cavity corresponding to the shape of the tooth in its centre, from two to three-eighths of an inch in depth, called the infundibula, better known as the cups in the teeth. Each incisor measures from two and half to three inches in length, and are firmly fixed in their sockets, their fangs or roots turned towards the centre of the jaw, the second pair folding over the centre or first pair, the third, or corner pair, folding in like manner over the second pair. These fangs or roots terminate in triangular points. The opposing surfaces vary in width and thickness as the animal advances in years, becoming narrower from side to side and deeper from before backwards, hence more triangular from year to year. To persons familiar with the anatomical structure of the teeth, these alterations are certain indications of age in the horse, even where the marks cease to exist, and the infundibula or cups have been obliterated. The natural wear of the incisor teeth is at the rate of one-sixteenth of an inch each succeding year, so by a close calculation a very fair estimate of the age of an animal may be made. It must be borne in mind, however, that there is a very great difference in the shape of the teeth in different animals, the horse with a shelly tooth being more triangular at their roots than one that is deeper or thicker. A strict observance of this rule would prevent very many errors which occur every day, even with men who claim to be experienced horsemen. The canine teeth, like the incisors, have but one fang, which is curved from without inwards. In the young horse they are sharp, externally convex, internally presenting two grooves on their surfaces, one on either side, and elevated in the centre. As the animal advances in years, these grooves disappear, the sharp points wear off, and the tooth becomes a rounded cone. These teeth are fully developed in the horse, but rarely or never in the mare. The molar teeth are placed at some distance from the incisors. The upper molars are about four times larger than the incisors, and the lower ones about three times as large. At maturity they measure from crown to root, or fang, about three inches. The upper molars may readily be known by their five projections or elevations upon their surfaces, while the lower ones have but four.

(*To be continued.*)

## Figures and Tables

**Figure f1:**