# Erratum

**Published:** 1882-10

**Authors:** 


WE call the attention of our readers to an error at the foot of page 290. Instead of "From two to five years old" read "From four to five years old."

